# Diabetes and obesity: the role of stress in the development of cancer

**DOI:** 10.1007/s12020-024-03886-1

**Published:** 2024-06-03

**Authors:** Angelo Avogaro

**Affiliations:** Department of Medicine.(DIMED), Unit of Metabolic Disease, University of Padova University of Padova, Via Giustiniani 2, 35128 Padova, Italy

**Keywords:** Stress, Diabetes mellitus, Obesity, Cancer, Non-communicable chronic disease

## Abstract

Diabesity is a condition where an individual has both diabetes and obesity, which can lead to severe complications including cardiovascular disease, a leading cause of mortality. Recently, cancer has become a leading cause of excess hospitalizations, and both diabetes and obesity are associated with a higher risk of developing several types of cancer. In this review, we propose that chronic stress significantly increases this association. Managing diabetes and obesity is challenging as they both cause significant distress. The relationship between stress and cancer is interconnected, with anxiety and depression being common in cancer patients. Cancer diagnosis and treatment can cause lasting changes in the body’s neuroendocrine system, with stress causing an excessive release of catecholamines and prostaglandins in patients undergoing cancer surgery, which promotes the spread of cancer to other parts of the body. Furthermore, stress could significantly increase the risk of cancer in patients with diabetes, obesity, or both.

## Introduction

**Fig. 1:**
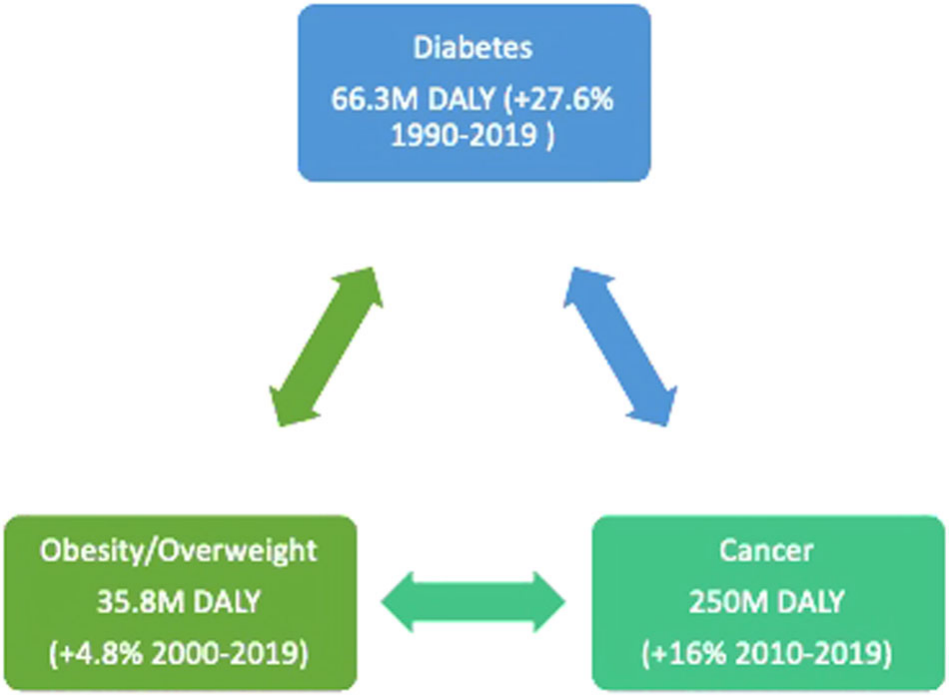
The changes of disability-adjusted-life-years (DALYs) Due to Type 2 Diabetes, Obesity, and Cancer (From: Global Burden of Disease World Health Organization https://www.who.int/data/gho/data/themes/mortality-and-global-health-estimates/global-health-estimates-leading-causes-of-dalys). Over the past 20–30 years, all three non-communicable chronic diseases have significantly increased DALY

Diabetes presents a profound challenge to global public health systems, affecting millions worldwide. The prevalence of this condition surged from 108 million in 1980 to 422 million in 2014, predominantly impacting low- and middle-income countries [1]. Diabetes can lead to severe complications such as kidney failure, heart attacks, stroke, blindness, and lower limb amputation, which can significantly impact the quality of life of those affected. The burden of Diabetes is substantial and contributes considerably to disability-adjusted life years (DALYs) as well as the increasing cost of healthcare (Fig. 1). In Italy, approximately 2.6 million patients receive drug therapies for Diabetes. The annual economic toll amounts to €20.3 billion, with indirect costs accounting for 54% [2].

Cardiovascular disease is a leading cause of mortality in diabetic individuals and increases the risk of coronary artery disease, heart failure, and stroke. In England, excess hospitalizations in 2003 were primarily due to diabetes and ischemic heart disease. However, by 2018, non-infectious respiratory conditions, non-diabetes-related cancers, and ischemic heart disease had become the leading causes of excess hospitalizations [3].

It is worth noting that more than 80% of individuals diagnosed with Diabetes are also obese. This highlights the link between these two major health issues. Obesity is a widespread public health problem that affects a significant portion of the American population [4]. The incidence of Diabetes has been following an upward trajectory similar to that of Obesity. Obesity disproportionately affects lower-income minority and disadvantaged groups, and it doubles the risk of developing type 2 diabetes. The cost of healthcare attributed to Obesity is expected to rise to 860.7–956.9 billion US dollars by 2030. This constitutes a significant portion of the total US healthcare expenses [5].

Although Grade 1 obesity may not increase mortality risk, higher BMI levels are linked to higher mortality rates, mainly due to cardiovascular disease and cancer [6]. It’s worth noting that in patients with “Diabesity,” there is a significant shift in the cause of death from cardiovascular disease to cancer. Therefore, it is crucial to have a deeper understanding of the underlying mechanisms to manage these health challenges effectively.

## Obesity and diabetes both contribute to cancer

A joint consensus statement released by the American Diabetes Association and the American Cancer Society has comprehensively evaluated the link between Diabetes and cancer [7]. The study reports that type 2 diabetes is associated with a higher risk of developing several types of cancer, including those affecting the liver, pancreas, endometrium, colon and rectum, and breast. This connection may be due to shared risk factors, such as aging, Obesity, dietary habits, and physical inactivity.

**Fig. 2:**
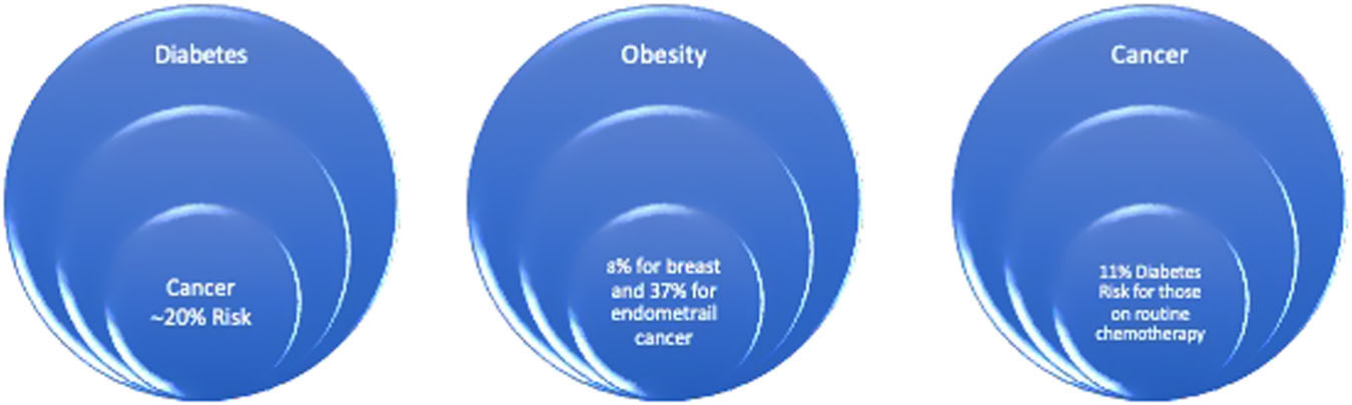
The relationships between diabetes and cancer, obesity and cancer, and cancer and diabetes

Cancer incidence is increasing in both type 1 and type 2 diabetes patients, regardless of the underlying causes [8]. A comprehensive meta-analysis has shown that the presence of type 2 diabetes is associated with a more than 20% increase in the risk of developing cancer [9]. A pooled analysis of 19 prospective Asian population-based cohorts showed that Diabetes was associated with a 26% increased risk of death from any cancer in Asians, emphasizing the need for better control of the growing epidemic of Diabetes as well as Obesity to reduce cancer mortality [10] (Fig. 2).

Additionally, patients with type 1 diabetes have a significantly higher incidence of cancer, particularly in the liver, pancreas, kidney, endometrium, and ovarian cancers [11]. Prediabetes is also linked to an increased risk of cancer, specifically in the liver, endometrium, stomach, and colorectal regions [12].

Since Diabetes is an independent risk factor for increased cancer-related mortality rates, it is crucial to ensure appropriate cancer screening for diabetic patients worldwide [13].

It is essential to highlight that managing Diabetes is possible in cancer patients. Various systemic anticancer therapies, such as cytotoxic chemotherapy, hormone therapy, targeted therapy, and immunotherapy, can affect glycemic control [14]. Several anticancer agents increase the risk of hyperglycemia, even in individuals without a prior diabetes diagnosis. A study has indicated that 11% of individuals undergoing routine chemotherapy received a new diabetes diagnosis, with most receiving short-course steroids alongside chemotherapy and 40% undergoing curative/adjuvant treatment [15].

Extensive research has been conducted to understand the relationship between metabolic conditions such as Obesity, hypertension, dyslipidemia, insulin resistance, and type 2 diabetes [16] and the risk of pancreatic cancer. The evidence suggests that there is a strong link between weight gain during adulthood and increased risks of various cancers, including post-menopausal breast, colorectal, endometrial, renal, and high-risk prostate cancers.

The EPIC and UK Biobank report underscores the strong correlation between Obesity and 13 different cancers, including breast, colorectal, endometrial, esophageal, pancreatic, renal, liver, stomach, gallbladder, ovarian, thyroid, multiple myeloma, and meningioma [17]. Moderate evidence suggests an association with cancers of the mouth, pharynx, larynx, prostate, male breast, and diffuse large B-cell lymphoma. Moreover, high risks are observed for challenging-to-treat cancers such as pancreatic, esophageal, and gallbladder, as well as prevalent malignancies like breast and colorectal cancer.

An umbrella review consisting of 204 systematic reviews and meta-analyses has highlighted an array of elevated cancer risks associated with excess body fat [18]. The risks range from 9% for rectal cancer in men to 56% for biliary tract system cancer. Every 5 kg/m^2^ increase in BMI leads to an increase in cancer risk [19]. It is important to note that for women who do not receive hormone replacement therapy, weight gain of 5 kg in adulthood increases the risk of post-menopausal breast cancer by 11%. On the other hand, the risk of endometrial cancer increases by 21% for every 0.1 increase in waist-hip ratio.

A Mendelian randomization study has indicated that there is a robust causal link between an increase in body mass index (BMI) and a higher risk of pancreatic cancer [20]. The study suggests that there is a 34% increase in pancreatic cancer risk for every standard deviation increase in BMI. It has also found a causal link between genetically increased fasting insulin levels and an increased risk of pancreatic cancer. However, there is no evidence to suggest a causal relationship between type 2 diabetes or dyslipidemia and pancreatic cancer. These findings indicate that BMI and fasting insulin may play a significant role in the development of pancreatic cancer. At the same time, type 2 diabetes or dyslipidemia may not be causally linked to it (Fig. 1).

In a study from Italy, researchers compared cancer incidence in populations with and without Diabetes, along with analyzing the excess risk of cancer associated with diabetes type, duration, and treatment [21]. The study cohort comprised 383,799 subjects without Diabetes and 23,358 with Diabetes; cancer incidence was calculated over a 4-year follow-up period. Overall, cancer incidence was higher in subjects with Diabetes, with specific sites such as the liver, pancreas, colon-rectum, and bladder showing increased risk, particularly in patients with type 2 diabetes. The analysis also indicated an escalating risk for diabetes duration up to 10 years from diagnosis, followed by a subsequent decrease to moderate-higher risk. Diabetes itself, insulin use, and diabetes duration were identified as potential factors influencing the association between Diabetes and cancer.

According to the most recent survey conducted in 2019, having a high body mass index (BMI) and high fasting glucose levels are considered to be among the most significant risk factors for cancer [22]. They caused a total of 133.9 age-standardized disability-adjusted life years (DALY) in that year. It is important to note that high fasting glucose and high BMI caused an increase in DALY in 2019 compared to the survey conducted in 2010.

## “Envirome,” diabetes, and obesity: they all produce stress

The environment, which encompasses all non-genetic factors, significantly impacts human health. It is estimated that 23% of global deaths and 22% of Disability-Adjusted Life Years (DALYs) result from environmental risk factors [23]. Non-communicable diseases (NCDs), such as Diabetes, Obesity, and cancer, are some of the tangible outcomes of an unfavorable environment. Recently, there has been an increased focus on the concept of the “exposome” or “envirome [24]” It refers to all the exposures a person encounters from conception to death, including natural, social, and personal environments. In this context, exposure to persistent organic pollutants [25] and endocrine disruptors [26] may specifically predispose to both Type 2 Diabetes and cancer. These interconnected layers can impact physical activity, nutrition, and mental stress.

When exposed to an unfavorable environment, a person’s body may experience a stress response in an attempt to maintain optimal function in challenging situations. This response is known as the “fight or flight” reaction, an automatic response to evolved threats [27]. However, this stress response can become chronic in an unfavorable environment and significantly negatively impact health [28].

The impact of stress on metabolism is more severe in diabetic patients than in control [29], and it depends on the prevailing glucose levels [30].

Regularly adapting to daily stressors can become a heavy burden known as the allostatic load, representing a chronic state of homeostasis at the expense of psychological and physical well-being. This dysregulated state involves the activation of neuroendocrine circuits, including the hypothalamus-pituitary-adrenal axis, sympatho-medullary axis, and autonomic nervous system.

Chronic stress has several consequences that affect the body in various ways [31]. For example, elevated levels of corticotropin-releasing hormone (CRH) and beta-endorphin can inhibit reproductive function by suppressing the release of gonadotropin-releasing hormone (GnRH). Chronic stress can also inhibit the GH-IGF-1 axis, suppress thyroid function, and disturb energy substrate metabolism, leading to insulin resistance. In addition, alterations in the immune response can occur, with glucocorticoids having an anti-inflammatory effect, while the autonomic nervous system can affect neutrophil demargination and cytokine production. All of these physiological changes, including insulin resistance, increased hepatic glucose production, disrupted sleep patterns, hyperlipidemia, and a maladaptive pro-inflammatory environment, collectively contribute to the pathogenesis of type 2 diabetes.

Furthermore, chronic stress can also hurt immune responses [32, 33]. Communication between the central nervous and immune systems occurs through shared chemical messengers, indicating a reciprocal regulatory relationship. Stressful conditions can lead to cellular-level alterations.

Diabetes, in itself, represents a distressful condition, and its intersection with chronic stress adds another layer of complexity to the intricate relationship between environmental factors and human health [34].

Many people who have Diabetes, whether it’s type 1 or type 2, face significant emotional challenges due to the demands of managing the disease and the potential for long-term complications. This emotional toll is called diabetes distress and is closely connected to the social aspects of living with Diabetes, such as stigma, discrimination, and the financial strain of the disease. If left unaddressed, mild diabetes distress can worsen and lead to severe distress and depression [35]. This emotional strain significantly affects the quality of life, particularly for women, as stress and diabetes distress can cause glycated hemoglobin levels to deteriorate.

Obesity comes with its unique stressors. People with higher weight status are at a higher risk of developing depression, and conversely, depression predicts future weight gain [36]. The psychological distress experienced by those who are obese is often worsened by weight stigma and discrimination, which can lead to feelings of devaluation and social marginalization. Consequently, individuals with higher body weight usually blame themselves for their situation, attributing it to overeating, especially indulgence in high-calorie comfort foods.

In summary, the interaction between the environment, Diabetes, and Obesity can cause significant distress, negatively affecting the affected individuals’ metabolic and immunologic responses. These factors work together to create a pro-oncogenic context, highlighting the complex relationship between emotional well-being, metabolic health, and the potential risk of developing cancer.

## Stress and cancer (mechanisms)

The relationship between stress and cancer is closely interconnected, with anxiety and depression being widespread in cancer patients [37]. The diagnosis and treatment of cancer can cause lasting changes in the body’s neuroendocrine system, with stress causing an excessive release of catecholamines and prostaglandins in patients undergoing cancer surgery, thereby promoting the spread of cancer to other parts of the body.

Several population studies have illuminated the correlation between chronic stress and cancer. While an association between workplace stress and the risk of prostate cancer was observed among Canadian men, it awaits further confirmation [38]. A meta-analysis of 142 prospective studies indicated a link between stress and an elevated incidence of lung cancer [39]. Subsequent analyses involving nine observational studies supported an association between work-related stress and risks for lung, colorectal, and esophageal cancers, albeit inconsistent with the findings [40]. Conversely, a prospective study involving UK women found no association between perceived stress levels or adverse life events in the preceding five years and the risk of breast cancer, a finding echoed by a similar study [41].

In contrast, a meta-analysis incorporating 12 cohort studies in Europe did not establish a link between work-related stress and the risk of lung, colorectal, breast, or prostate cancers [42]. Despite these varied outcomes, the overarching understanding is that psychological stress can contribute to the incidence, recurrence, and progression of cancer. This multifaceted relationship underscores the need for continued research and a nuanced understanding of the interplay between stress and various cancer types.

Stress complexly impacts the body’s physiology by activating systems such as the sympathetic adrenal-medullary (SAM), hypothalamic-pituitary-adrenal (HPA) axes, and the peripheral and parasympathetic nervous systems. This complex network changes the release of molecules that affect the immune system in response to stress, including catecholamines, cytokines, and glucocorticoids [43]. These changes can significantly impact the immune environment within tumors.

The increase in glucocorticoids has various effects. It weakens the priming of antigen-specific cytotoxic T lymphocytes (CTLs) by reducing CD8+ dendritic cells, which can promote radiation-induced tumorigenesis by activating the serum/glucocorticoid regulated kinase 1 (SGK1) and the E3 protein ubiquitin Mouse double minute 2 ligase (MDM2) [44]. In the case of breast cancer cells, glucocorticoids activate the transcription factor (TEAD)4, which in turn promotes tumor growth and metastasis [45]. Stress-induced glucocorticoids intensify the metastatic colonization of breast cancer cells by increasing the expression of receptor tyrosine kinase-like orphan receptor 1 (ROR1) and its ligand WNT5A [46]. Additionally, glucocorticoids contribute to cell death by increasing the production of reactive oxygen and nitrogen species, which cause DNA damage and impair repair mechanisms. Finally, they also promote cancer stemness and chemoresistance by activating TEA Domain Transcription Factor 4 (TEAD4) and YAP [47].

Both acute and chronic stress significantly affect the dopaminergic system, triggering dopamine release and influencing various immune responses, including the stimulation of naive CD8 + T cells and inhibition of natural killer cell expansion and interferon-gamma production [48]. Integrating stress responses, noradrenaline, and adrenaline contribute to tumor initiation, progression, dissemination, and therapeutic resistance. They induce excessive DNA damage and genomic instability by triggering TP53 degradation, elevate lactate dehydrogenase A (LDHA) in breast cancer cells, and exert an anti-apoptotic effect on prostate and breast cancer cells [49].

Furthermore, stress-induced catecholamines may influence the cancer environment, impacting tumor progression and metastasis through neurotransmitters like substance P and acetylcholine [50]. The density of sympathetic and parasympathetic nerve fibers in tumors has been linked to poor clinical outcomes. Stress can also impact stromal components, stimulating the secretion of inhibin-βA (INHBA) from cancer cells and activating cancer-associated fibroblasts, leading to collagen deposition and extracellular matrix (ECM) formation [51].

Stress hormones can trigger the production of cyclooxygenase COX-2 and the COX-2/PGE2 pathway, which cause inflammation and affect the tumor microenvironment [52]. The metabolic patterns that stress induces can cause immune cells to mobilize glucose and lipids, resulting in a metabolic shift that affects tumor responses [53]. Chronic stress can weaken T cell glycolysis and oxidative phosphorylation, leading to T cell exhaustion in the tumor microenvironment. Regular stress-induced adrenaline can activate LDHA upregulation, promoting glycolysis in mammary tumors.

**Fig. 3:**
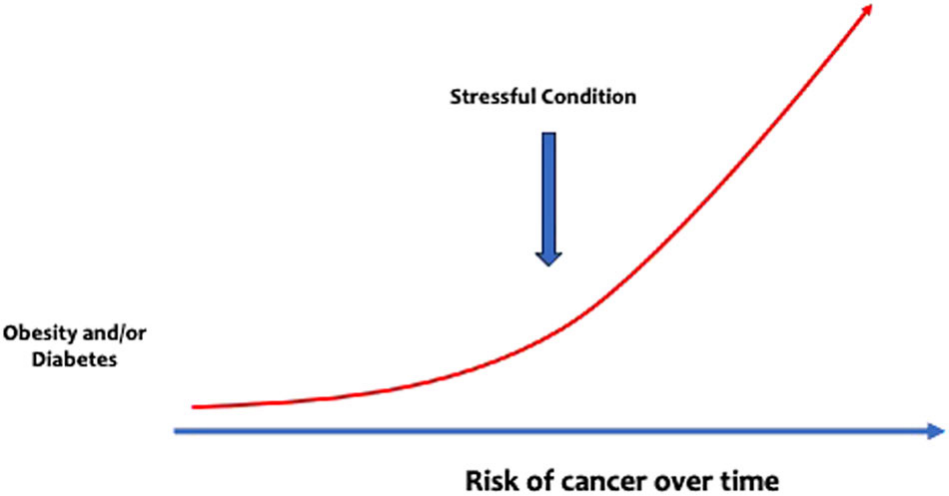
Chronic stress is per se able to induce cancer; however, in patients either with diabetes or obesity or both, stressful condition can dramatically increase the risk for cancer

In conclusion, chronic stress triggers the hypothalamic-pituitary-adrenal (HPA) axis and the sympathetic nervous system (SNS), which can result in DNA damage and activation of pro-oncogenic intracellular pathways. Stress also impacts the microenvironment, affecting tumor growth, invasion, and metastasis. The complex interplay between stress, immunological, neuronal, and metabolic responses highlight the significant influence of stress on cancer progression. Moreover, stress could dramatically increase the risk of cancer in patients either with diabetes or obesity or both (Fig. 3).

## Obesity and cancer (mechanisms)

Obesity is associated with type 2 diabetes mellitus, cardiovascular disease, hypertension, and nonalcoholic fatty liver disease. Adipose tissue possesses an extraordinary endocrine machinery, which can be modulated by either energy excess or starvation [54]. In obese people, elevations in circulating leptin, insulin, and insulin-like growth factor (IGF)-1 can each contribute to the excess cancer risk [55]. Leptin, produced by adipose tissue proportionally to its mass, regulates the immune response: humans with mutations in the leptin gene have CD4^+^ T cell immunodeficiency and, therefore, an increased risk of infections [56]. Leptin also influences B cells and innate immune cell responses. Insulin is also a key regulator of immune cells, which can modulate immune cell differentiation and function, particularly CD4^+^ T cells. Similar to leptin, the effects of insulin on T cell function are mediated through changes in cellular metabolism via regulation of glucose uptake and amino acid transport, with increased mitochondrial respiration [57]. IGF-1 affects T cell development and function: it has been identified as a potent regulator of TH17 cells, a subset of pro-inflammatory T helper cells that produce interleukin (IL)-17 function [58]. IGF-1 increases mitochondrial oxidation in CD4^+^ T cells, particularly TH17 cells. In contrast with these observations, calorie restriction decreases tumor growth in mouse models, attributed to reduced insulin and IGF-1 levels and enhanced tumor immunity [59].

Adipose tissue can directly affect tumor development and progression, such as in breast cancer, where milk glands are embedded in breast adipose tissue, but also in pancreatic, kidney, melanoma, and prostate cancers [60]. This interaction is bidirectional. Tumor cells can deplete adipocytes of lipids; this effect leads to the dedifferentiation of adipocytes into myofibroblasts, mesenchymal stem cells, and macrophage-like cells, capable of supporting tumor cell proliferation and invasion. Specifically in breast cancer, adipose tissue leads to the growth and progression of cancer not only via the increased local production of adipokines and inflammatory mediators but also by supporting the energetic demands of cancer cells, such as increased vascularization or release of free fatty acids (FFAs). Clinically, growth factor signaling pathways, such as VEGF and AkT, are increased in the breast tissue of obese adolescents relative to non-obese adolescents [61]. In this context, the highest breast cancer risk has been reported for women with breasts in the highest quintiles for fat tissue [62].

Interestingly, the role of exosomes has also been recently emphasized: they are derived by the adipocytes, probably as a consequence of hypoxia, and they can contain nucleic acids, proteins, and metabolites [63]. Adipose tissue can release 1% to 2% of its lipid content via exosomes: their content may have critical biological roles via cargo delivery. Exosomes stimulate the proliferation and metastasis of breast cancer cells [64]. Extracellular vesicles from breast adipose tissue of women with Obesity induce breast cancer cell proliferation by increasing mitochondrial density and mitochondrial oxidative phosphorylation. Exosomes from adipose-derived mesenchymal stem cells also stimulate breast cancer cell migration with potential mechanisms involving activation of Wnt and Hippo signaling pathways [65].

In patients with Obesity, alterations in intermediary metabolism, especially of lipids, play an important role in predisposing these subjects to many cancers, including prostate cancer. Fatty acids and their incorporation into the plasma membrane are required for cell division, yet they are also the primary fuel source for some tumor types, such as prostate cancer [66]. Given that dyslipidemia and intake of saturated fatty acids are associated with an increased recurrence and risk of mortality in men with prostate cancer, these substrates contribute to supporting the growth of prostate cancers and driving disease progression [67]. Following this observation, the fatty acid transporter CD36 was positively associated with disease recurrence in men. Similarly, expanded adipose tissue may negatively affect other types of cancer, such as melanoma [68], kidney cancer [69], and multiple myeloma [70]. In conclusion, several studies demonstrate a strong association between Obesity and cancer, and most of the pathogenetic links between the two diseases have been consistently clarified. However, the role of adipocytokines needs to be further clarified.

## Diabetes and cancer: pathogenetic links

Several studies highlight the increased risk for cancer in patients with type 2 diabetes and involve multiple factors that frequently coexist [71]. High insulin and blood glucose levels, the hallmarks of patients with Diabetes, serve as growth factors for cancer development. Hyperglycemia induces oxidative stress and DNA damage, initiating tumorigenesis. Epidemiological data link glycated hemoglobin levels to increased risks of colorectal, gastric, and pancreatic cancers [72–74]. In vitro studies show that high glucose concentrations alter gene expression, promoting proliferation, migration, and invasion in cancer cells [75]. Hyperglycemia may also generate advanced glycation end products and inflammation, promoting tumor transformation and resistance to oxidative stress. ROS disrupts intracellular signaling associated with antitumoral activity and leads to the inactivation of Phosphatase and tensin homolog (PTEN), increasing PI3K/Akt signaling that promotes proliferation [76, 77].

Oxidative stress and inflammatory response are always linked to each other in the tumor progression. One of the primary contributors to the development of tumors is a molecule called nuclear factor-κB (NF-κB). It plays a crucial role in cells and is highly sensitive to oxidative stress, being able to detect even low levels of hydrogen peroxide (H2O2). Activating mitogen-activated protein kinases (MAPKs) by reactive oxygen species (ROS) leads to the production of inflammatory mediators, activating NF-κB. Once activated, NF-κB helps to suppress cell death and stimulates cell proliferation [78]. Oxidative stress, NF-kB, and JAK-STAT pathways work together in cancer progression. ROS also directly affects cell structures: ROS interacts with cellular macromolecules such as DNA, proteins, and lipids, interfering with vital cellular functions. They lead to DNA base alterations, strand breaks, damage to tumor suppressor genes, and expression of proto-oncogenes, transforming normal cells into malignant cells [79].

Endogenous hyperinsulinemia, common in type 2 Diabetes due to reduced insulin sensitivity, activates insulin receptors, IGF-1 receptors, and hybrid insulin/IGF-1 receptors, promoting cancer cell proliferation, survival, and metastasis [80]. High insulin levels correlate with increased risks of kidney, bowel, colon, and breast cancers. Insulin’s role in cell proliferation is well-documented, significantly elevated when insulin receptor expression leads to enhanced proliferation and loss of cell contact inhibition. Obesity intensifies this environment, as adipocytes from obese individuals secrete higher levels of IGF-1. It is well known that patients with acromegaly present elevated IGF-1 levels, which increase the risks of colon, breast, thyroid, and prostate cancers [81]. IGFs promote tumor growth, migration, and invasion through paracrine and autocrine routes. Insulin receptor signaling activates mitogenic PI3K/AKT/mTOR, and MAPK/ERK signaling cascades frequently mutated during tumorigenesis.

Interestingly, the mechanisms underlying the tumorigenesis of insulin in pancreatic cancer have been recently shown [82]. The insulin receptor in acinar cells is causal in supporting cancer initiation in diet-induced Obesity. The presence of increased production of enzymes mediated by high insulin concentrations raises the risk of a more autoactivated trypsin as well: this results in a trypsin-induced injury and a concomitant sub-clinical inflammation and acinar-to-ductal metaplasia. These data indicate that hyperinsulinemia, acting through the insulin receptor (Insr), is the upstream driver of diet-induced inflammation via hyperactive digestive enzyme production.

Glucose in the microenvironment significantly increases the number of cancer stem cells (CSC). In leukemia, stem cells are predominantly powered by oxidative phosphorylation (OXPHOS), while their differentiated progeny rely on aerobic glycolysis for energy supply [83]. These stem cells depend on glycolysis and show highly expressed glucose transporters, hexokinase, and pyruvate dehydrogenase. This implies that different types of CSCs have different pathways of glucose metabolism [53]: compared with OXPHOS, aerobic glycolysis produces lower ATP but at a faster rate. Aerobic glycolysis consumes 1 molecule of glucose to yield 2 mol of ATP. In comparison, complete oxidation of 1 glucose molecule produces 32 mol of ATP, but aerobic glycolysis produces ATP approximately 100 times faster than OXPHOS. When CSCs drive the massive expansion of tumor cells and the demand for ATP is significantly increased, rapidly enhanced aerobic glycolysis is conducive to maintaining some CSCs’ stemness characteristics, and a substantial number of metabolic intermediates favorably cater to rapid cell proliferation [84].

The large production of lactate during aerobic glycolysis affects the cellular and cytokine composition of the tumor microenvironment, which in turn promotes tumor development by influencing multiple other pathways and mechanisms [85]. The relationship between glucose and cancer is substantiated also by immunometabolism, i.e., how immune cells employ nutrients to support their growth and functionality [86]. Tumor-associated macrophages are present in large numbers in solid tumors and play a crucial role in regulating tumor growth. They suppress the immune system and facilitate immune evasion through mechanisms such as TGF-β and IL-10. They also promote the formation of new blood vessels (angiogenesis) by secreting vascular endothelial growth factor (VEGF) and protect tumors from oxidative stress, thereby mediating resistance to chemotherapy. In addition, they promote tumor growth after radiation [87].

Potential effectors of tumorigenesis caused by glucose are microbiome and epigenetics. The dysbiotic configuration reported in the microbiome of Diabetes patients led to the onset and development of chronic low-grade inflammation [88]. Inflammatory cytokines and NADPH oxidase are overexpressed in the colon tissues of diabetic mice. Cazzaniga and colleagues have reported how the disruption of the Firmicutes/Bacteroidetes ratio in gut microbiota contributes to increasing cancer risk and indirectly increasing the onset of metabolic disorders, including insulin resistance, metabolic syndrome, and Diabetes [89]. Specifically, the reduction in short-chain fatty acids (SCFAs)-producing bacteria is widely associated not only with metabolic disorders, including DM and Obesity, but also with different cancer types.

Evidence supports that hyperglycemia can cause epigenetic chromatin changes in target cells, leading to sustained epigenetic modifications of gene expression profiles in human cells [90]. Histone proteins are highly prone to glycation due to their essential nature and long half-lives: the consequences of these effects are the histone-histone and histone-DNA cross-linking, resulting in alterations of the dynamic architecture of chromatin. Hyperglycemia can directly rewire the epigenome towards an oncogenic state: hyperglycemia abolishes the AMPK-mediated phosphorylation of the tumor suppressor (TET2) [91]. Alteration of TET functions is reported in many cancer types. In addition to DNA methylation and chromatin modifications, noncoding RNAs (miRNA) are significant contributors to epigenetic regulatory mechanisms [91]. miRNAs are also involved in the initiation and progression of cancers. Some miRNAs in the context of DM can impact cellular processes involved in carcinogenesis, such as cell proliferation, angiogenesis, and epithelial-to-mesenchymal transition (EMT) [92]. For instance, miR-93 is a miRNA that regulates VEGF, a crucial factor in tumor angiogenesis and microvascular complications in DM [93]. At the same time, miR-375 is involved in insulin secretion and beta-cell function and is implicated in the proliferation of human cancer cells. Interestingly, there are critical pathways linking Diabetes and cancer: (1) mitogen-activated protein kinase (MAPK) signaling pathway, (2) INSR signaling cascade (KEGG), (3) mammalian target of rapamycin (mTOR) signaling pathway, (4) PI3K cascade, (5) phosphatidylinositol signaling system, and (6) apoptosis [94]. In all these pathways, a significant participation of miRNA was found; therefore, miRNAs should be considered not only a link between Diabetes and cancer but can represent a therapeutic target.

## Conclusions

**Fig. 4:**
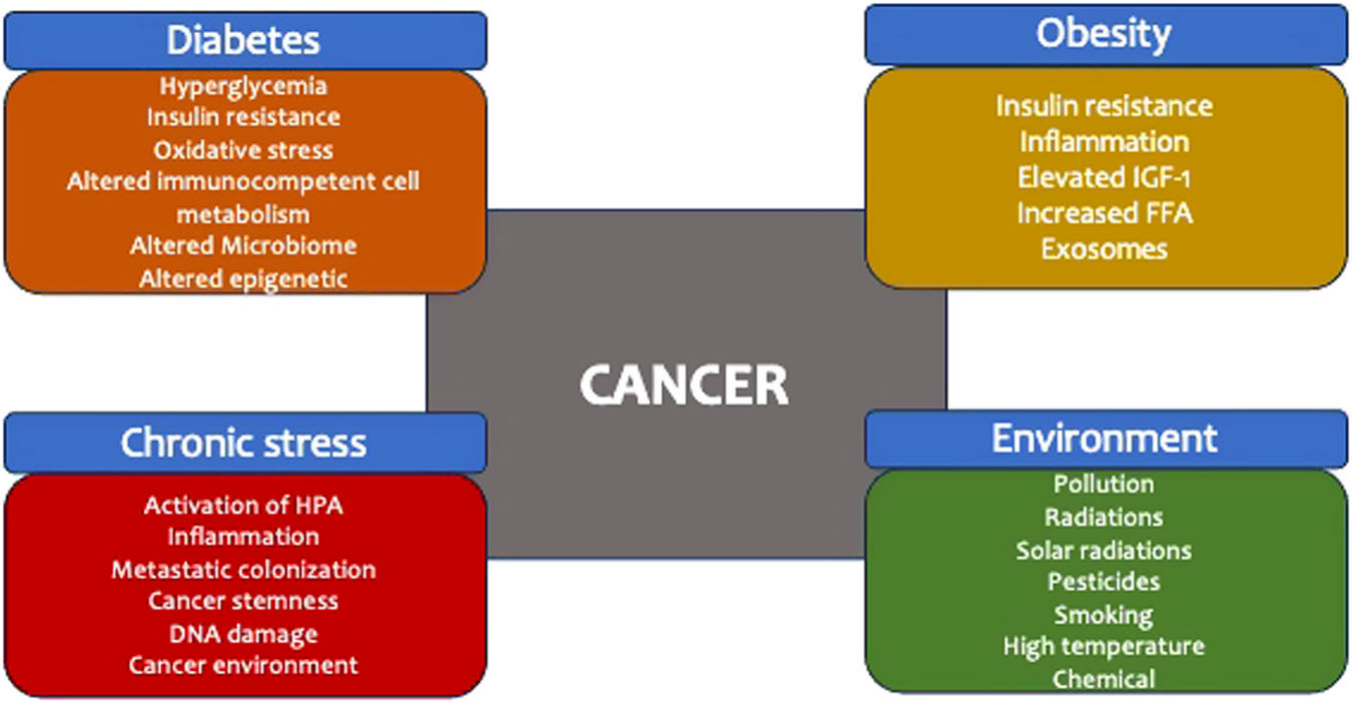
Obesity, diabetes, stress, and environment can increase the risk of cancer, each through specific alterations in cellular homeostasis and cancer environment

Diabetes and Obesity are two of the most common non-communicable chronic diseases that significantly impact people’s health by causing short and long-term complications. These conditions also create significant distress, making their management more difficult. People who have Diabetes and Obesity are exposed to a stressful environment. In this article, we have discussed how Diabetes and Obesity can increase the risk of cancer. We have also highlighted how stress, along with an unsafe environment, can multiply the cancer risk in patients with metabolic disease (as shown in Fig. 4). While stress is unavoidable, reducing the risk of cancer can be achieved by maintaining metabolic control, following a healthy diet, engaging in regular exercise, improving sleep quality, and losing weight [95].
